# Validation of Two Instruments for the Measurement of Dehumanization and Self-Dehumanization in Healthcare Settings

**DOI:** 10.3390/nursrep14030167

**Published:** 2024-09-06

**Authors:** Aikaterini Roupa, Athina Patelarou, Evangelos C. Fradelos, Kyriaki Fousiani, Marianna Miliaraki, Konstantinos Giakoumidakis, Evridiki Patelarou

**Affiliations:** 1 Department of Nursing, School of Health Sciences, Hellenic Mediterranean University, 71410 Heraklion, Greece; apatelarou@hmu.gr (A.P.); kongiakoumidakis@hmu.gr (K.G.); epatelarou@hmu.gr (E.P.); 2 Department of Nursing, School of Health Sciences, University of Thessaly, 41500 Larisa, Greece; efradelos@uth.gr; 3 Department of Psychology, University of Groningen, 9712 Groningen, The Netherlands; k.fousiani@rug.nl; 4 Pediatric Intensive Care Unit, School of Medicine, University of Crete, 70013 Heraklion, Greece; med1p1130027@med.uoc.gr

**Keywords:** animalistic and mechanistic dehumanization, hetero- and self-dehumanization, healthcare, doctor, nurse, hospital

## Abstract

Understanding and addressing dehumanization in healthcare is crucial due to its profound impact on patient care, ethical implications on patient dignity and autonomy, and its potential to affect the psychological well-being of healthcare professionals. The primary aim of this study was to establish reliable and valid instruments measuring two different types of dehumanization, namely animalistic dehumanization (i.e., stripping one of their uniquely human characteristics) and mechanistic dehumanization (i.e., stripping one of their human nature characteristics) among healthcare professionals. In this cross-sectional validation study among healthcare professionals, we tested measures of both animalistic and mechanistic dehumanization, focusing on the dehumanization of patients (hetero-dehumanization) and oneself (self-dehumanization), respectively. All measures were developed and validated based on a concept analysis, a literature review, and an appraisal of pre-existing scales. The research was conducted among 400 nurses and medical doctors employed in Greek public hospitals. Coefficient validity ratio results showed that 100% of items were acceptable for both measures. The newly established and validated hetero-dehumanization scale encompassed two factors (factor 1: animalistic dehumanization, factor 2: mechanistic dehumanization; Cronbach’s alpha was equal to 0.86 for each measure). The self-dehumanization scale was a mono-factorial measure of mechanistic dehumanization (Cronbach’s alpha = 0.97). Two validated measures of (self- and hetero-) animalistic and mechanistic dehumanization measures were developed for the assessment of dehumanization among health professionals, which will form the basis for future research in this important scientific field.

## 1. Introduction

Dehumanization is a concerning phenomenon that involves perceiving certain individuals as less than human, thereby stripping them of their inherent human qualities and rights, and it contributes to escalating discrimination and mistreatment. Specifically, dehumanization manifests as the tendency for some individuals to deny others their humanity (hetero-dehumanization), depriving them of fundamental rights such as respect and equity [1,2], as well as themselves (self-dehumanization), as a coping mechanism to shield themselves from emotional distress or to meet societal norms or expectations. This phenomenon concerns both interpersonal and intergroup relationships and is often accompanied by violence or aggression. As such, dehumanization involves socio-cognitive aspects and is associated with moral exclusion and moral disengagement [2,3,4,5].

### 1.1. Hetero-Dehumanization

The dehumanization literature identifies two types of hetero-dehumanization, namely animalistic and mechanistic dehumanization [2,3]. Animalistic dehumanization involves the reduced attribution of exclusively human qualities (human uniqueness) to others (e.g., logic, morality, sensitivity, kindness), thus considering people as driven by impulses, moods, and instincts similar to those of animals. As a result, animalistically dehumanized individuals are treated with disgust, contempt, and acts of humiliation. Mechanistic dehumanization involves stripping individuals of their inherent human qualities (human nature), such as emotional responsiveness, interpersonal warmth, and cognitive receptivity, which are fundamental aspects of their humanity.

Accordingly, mechanistically dehumanized human beings are treated as machines or objects and are considered to lack individual identity. As a result, mechanistically dehumanized targets are viewed as introverted and passive beings unworthy of moral concern or care [6,7,8].

Hetero-dehumanization, be it animalistic or mechanistic, is particularly relevant among healthcare professionals due to its potential impact on patient care, communication, trust, and health disparities [9]. When healthcare providers perceive patients as subhuman beings (i.e., animals) or objects rather than as autonomous entities with emotions, preferences, and rights, it can result in negligence, dismissiveness, or even mistreatment, thereby compromising the quality of care provided [10]. For instance, modern medicine often relies on standardized biomedical models, neglecting personalized care tailored to individual needs and emotional support. According to Lebowitz and Ahn (2014) [11], when physicians focus on the biological and genetic explanations for diseases, they can perceive patients as physiologically different from “healthy” people. Consequently, this perspective can diminish empathy and foster dehumanizing behaviors in healthcare settings [11], leading to the treatment of the body as a machine or object and illness as a malfunction of body mechanics. Institutions such as hospitals even favor such latent forms of dehumanization due to their structure and organization [12].

### 1.2. Self-Dehumanization

As previously stated, besides dehumanizing others, people often dehumanize themselves as well, which they do as a coping mechanism to protect themselves from emotional pain or to conform to social expectations [9]. Indeed, although self-dehumanization is associated with aversive self-awareness, lack of self-esteem, or a meaningful life, and with the experience of intense negative feelings (such as shame, sadness, anger, and guilt), people may resort to self-dehumanization processes to mitigate the impact of witnessing others’ suffering [1,13]. More specifically, research shows that when employees are continuously exposed to stressful work environments, such as the healthcare environment, they tend to dehumanize themselves, an act that serves as a defensive mechanism that medical staff use to protect themselves from negative feelings. Unsurprisingly, self-dehumanization is related, in turn, to treating patients as objects rather than autonomous human beings [13]. In a similar vein, Trifiletti et al. (2014) [9] suggested that animalistic dehumanization of patients is a way for nurses to cope with work-related stress and stress-related symptoms [9,14].

Interestingly, previous research shows that when it comes to self-dehumanization, individuals tend to exhibit more mechanistic rather than animalistic dehumanization. In essence, people are hesitant to strip themselves of their uniquely human qualities, yet they are inclined to disassociate from their human nature characteristics when confronted with emotionally challenging circumstances. This phenomenon can be attributed to two primary factors: First, animalistic dehumanization may not offer any adaptive advantages to individuals as it fails to serve as a defense mechanism. Secondly, animalistic dehumanization could be linked to a decrease in social standing, leading to feelings of inferiority that are detrimental to individuals’ well-being [15,16].

### 1.3. The Current Study

Despite its prevalence in healthcare settings, dehumanization remains inadequately explored, with a notable absence of validated questionnaires tailored to assess dehumanization within the healthcare domain. Given the emotionally taxing nature of healthcare, characterized by suffering and pain [14,17], we argue that validated measures specifically addressing dehumanization in this context are imperative. Furthermore, it is crucial to recognize that dehumanization is profoundly influenced by cultural and broader social factors [18]. Indeed, in contexts such as countries where healthcare institutions lack resources or infrastructure, both professionals and patients may encounter significant challenges. Greece exemplifies such a context, where healthcare professionals grapple with substantial difficulties in navigating the inherent suffering within the healthcare system. Therefore, investigating dehumanization among healthcare personnel in Greece presents an intriguing opportunity to explore the interplay of cultural dynamics, systemic challenges, and social contexts within the healthcare system. Greece’s economic crisis and the influx of migrants and refugees may have contributed to the increased levels of dehumanization among healthcare providers [19].

In this context, everyday dehumanization is believed to occur largely unconsciously and unintentionally as a result of various factors within the healthcare system. In other words, people can dehumanize others as an emotional regulation strategy, thereby avoiding feeling emotionally drained and overwhelmed by witnessing the pain of others [20]. An important causal factor, beyond personality and personal experiences, is burnout, the professional syndrome of exhaustion, depersonalization, and lack of meaning, which has increased from 40% to 60% among healthcare professionals over the past four decades [21]. This high prevalence creates a healthcare crisis by diminishing productivity, quality of care, and job satisfaction. It also leads to more medical errors and higher job turnover rates, negatively impacting the physical, emotional, social, and spiritual well-being of healthcare professionals [21,22,23]. In the context of the latest financial crisis, burnout has become a particularly crucial parameter of depersonalization for Greek healthcare workers, with recent reports of high burnout scores in more than 40–75% of the participating healthcare professionals [24,25,26,27].

The research gap is significant in highlighting the phenomenon, and its importance is considerable for humanity. In a study conducted on medical students, it was revealed that inequalities in healthcare are a widespread form of minority discrimination in medical contexts. Racist interactions represent 75% of interactions in the healthcare sector in the USA. During racially discordant interactions, cognitive mechanisms such as implicit bias and racial prejudice of doctors have a huge impact, affecting patient satisfaction, perception of teamwork, and treatment adherence [28].

Another study focused on the provision of healthcare services after sexual assault. A significant issue was highlighted as scientists revealed the depersonalization experiences of survivors. These experiences included feelings of rejection, shame, and guilt, taking into account the needs and experiences of African American women who survived sexual assaults [29].

The behaviors of doctors in ICUs can make patients and their families feel dehumanized when they are in the ICU. Negative behaviors are observed by patients and families, possibly contributing to poor outcomes such as mental health, recovery, and lack of trust in ICU teams [30].

Other researchers examine dehumanization as it manifests towards individuals with disabilities. The individuals with cognitive disabilities are perceived as parasites to the social body [2]. They are considered incapable of living civilized lives and insensitive to pain, while there is a perception that they tend towards immoral and criminal behavior. In this context, mental illness can be seen as a type of cognitive disability. Individuals with mental illnesses are stigmatized and dehumanized, which contributes to the maintenance of disorders [31,32].

Some nurses stigmatize and dehumanize individuals with psychiatric disorders more than those without psychiatric disorders [20]. It was also found that some patients with organic disease and patients with psychosis do not engage in mechanistic and animalistic dehumanization of themselves. However, it appears that insecure attachment (anxiety and obsession) positively contributes to their mechanistic dehumanization and negatively to their mechanistic self-dehumanization. Regarding patients, it seems that patients with psychotic disorders attribute fewer characteristics of human uniqueness, apparently due to the cognitive disorders caused by the disease, stigma, and self-stigmatization [33,34].

On the other hand, it has been reported that dehumanization might, to some extent, benefit healthcare professionals, facilitating pro-social behaviors in medical contexts. However, this is limited to a very specific context and requires further research to be substantiated in the future [35].

Accordingly, understanding the prevalence and implications of dehumanization among healthcare providers in this context can inform efforts to enhance professional training, organizational support systems, and patient-provider relationships, ultimately contributing to the improvement of healthcare quality, equity, and compassion in Greece and beyond.

Thus, the main aim of the present study was the validation of appropriate tools measuring both forms of dehumanization, animalistic and mechanistic, targeted towards patients (hetero-dehumanization) and to oneself (self-dehumanization), among healthcare professionals, including doctors and nurses working in public hospitals in Greece.

## 2. Materials and Methods

### 2.1. Participants

The research was conducted in 6 public hospitals in Crete (Greece), and data were collected between November 2021 and January 2022. A convenience sample of 400 medical doctors and clinical nurses was recruited in total. With regard to the healthcare departments participating in the present study, we defined the closed departments of a hospital (e.g., closed intensive care units) as units, whereas wards refer to open clinical departments (e.g., the pediatric or the pathology department). When it comes to working hours, cyclical hours refer to early, late, and night rotating shifts, whereas shifts refer only to non-rotating shifts.

### 2.2. The Questionnaires

We used the hetero-dehumanization scale developed by Fousiani et al. (2019), which consisted of 12 items and was organized in two subscales: animalistic hetero-dehumanization and mechanistic hetero-dehumanization [36]. This scale, which was based on Haslam’s (2006) [2] dehumanization model, has not been used among healthcare professionals and has not been validated. The items were measured on a 7-point Likert scale (1 = absolutely disagree, 7 = absolutely agree) [36,37,38]. A sample item for animalistic hetero-dehumanization was “(7) They are rational and sensible people. They are smart; (8) They are persons inferior to the human species, like animals” (Appendix A). A sample item for mechanistic hetero-dehumanization was “(1) They are superficial people and have no depth; (2) They are open-minded people who can think clearly” (Appendix B).

We also used a self-dehumanization scale consisting of 10 items measuring dehumanization among healthcare personnel, which was developed by Sakalaki et al. (2016, 2017) but was not validated [1,2,6,16]. The scale exclusively focused on mechanistic self-dehumanization, as prior research indicates that individuals are inclined to show a greater tendency towards mechanistic rather than animalistic dehumanization. The items were measured on a 9-point Likert scale (1 = absolutely disagree to 9 = absolutely agree). A sample item was “(1) I make most of my decisions based on logic. (2) I don’t usually operate in an emotional way”.

Participants received the questionnaires after the researcher personally approached them in the departments where they worked. They were informed that the research concerned hospitalized patients, without providing further details to avoid bias. It was also pointed out that participation in the research was voluntary and anonymous, and that there were no right or wrong answers. The questionnaires distributed to the participants were accompanied by the explanatory text. Participants completed their socio-demographic characteristics, including some work-related questions. The average time to complete the questionnaire was 10 min. The questionnaire was evaluated by three experts in the field of dehumanization, who therefore had the experience and expertise to evaluate the tool and to propose methods for improving it (content validity) [39]. Items with a Coefficient Validity Ratio (CVR) > 0.70 were preserved in the final version of the instrument. We also calculated the content validity index (CVI) for the instrument as a whole, considering a value > 0.80 to be adequate. Afterward, a group of five doctors and five nurses was invited to test the scale for its linguistic precision and clarity and confirm that the scale consists of questions that are consistent with the attribute to be measured and do not result in an incomplete response to the questions or misleading answers [40]. These individuals were excluded from the final study’s sample.

### 2.3. Ethical Considerations

This survey was carried out in full compliance with the new General Data Protection Regulation (GDPR) [EU 2016/679] on 25 May 2018 on sensitive personal data. Prior to its implementation, the relevant approval was secured by the respective services. The present report was approved by the Hellenic Mediterranean University of Heraklion, Crete, Greece (Approval Code: 55/EMP (87)/24 May 2021). The data collected were anonymous, their use was made solely for the purposes of the survey, and access to them by the lead researcher. The participants gave their informed consent prior to the data collection.

### 2.4. Analytical Plan

We performed the statistical analysis using SPSS version 26.0 (SPSS Inc., Chicago, IL, USA). To check the condition of normality, the Shapiro-Wilk test was used along with the study of the graphic representations “Normal Q-Q plot”, “Detrended Normal Q-Q plot”, and “Box Plot”. For all tests, statistical differences were determined to be significant at *p* < 0.05. Continuous variables were represented as mean ± standard deviation and categorical variables as frequency and percentage. The relationship between two continuous variables, the Pearson coefficient, between a continuous and an ordinal variable, the Spearman correlation coefficient, and between a continuous and a dichotomous variable, the Point Biserial coefficient, was investigated. Analysis of variance (ANOVA) was used to study the relationship between a continuous and a nominal variable. Internal consistency was assessed by Cronbach’s alpha. A Cronbach’s α coefficient of >0.7 indicates sufficient reliability for research purposes and suggests that items are interdependent and homogeneous in terms of the construct they measure. For clinical applications, α > 0.8 is desirable. Exploratory Factor Analysis, using the principal component extraction method with Varimax rotation, was conducted to determine the factor structure of the items of the (hetero) dehumanization and self-dehumanization scales. Bartlett’s test of sphericity was conducted to examine the correlation among the items. The Kaiser–Meyer–Olkin (KMO) measure of sampling adequacy was computed for quantifying the degree of intercorrelations among the variables and the appropriateness of factor analysis. To justify factor analysis, KMO values should exceed 0.60. For the final model, we used the combination of the following selection criteria: (a) sample size ≥ 250, (b) scree plot, (c) each factor contains items with loading ≥ 0.40 while at the same time loading < 0.40 to all other factors, (d) each factor contains at least three items with loading ≥ 0.40, and (e) proportion of the total variance explained by the retained factors should be at least 60%.

## 3. Results

The sample comprised 400 participants, 297 (74.3%) females and 103 (25.7%) males, with a mean age of 43.8 years (Table 1). Further, 54.5% of them lived in a city with >150,000 inhabitants, 31.8% in a city with <150,000 inhabitants, and 13.8% in a village; 59.3% were married, 32.8% were single, 7.3% divorced, and 0.8% widowed. In this study, 73.5% of the participants were nurses, and 26.5% were medical doctors. With regard to those healthcare professionals with higher academic qualifications, 16.5% hold a master’s degree and 3.7% a PhD. However, with regard to the whole sample of the study, around 79.8% of healthcare workers do not hold a postgraduate diploma; 51.3% worked in unit sections and 48.7% in the wards; 74.5% worked in cyclical hours and 25.5% in shifts. The average working time in the department was 9.2 years. Psychotherapy sessions were attended by 16.5%. Table 2 and Table 3 list in detail (absolute number and percentage) the responses given to each question in the questionnaires of hetero- and self-dehumanization.

### 3.1. Animalistic and Mechanistic Hetero-Dehumanization and Self-Dehumanization Scales

#### 3.1.1. Animalistic and Mechanistic Hetero-Dehumanization Scale

**Figure 1:**
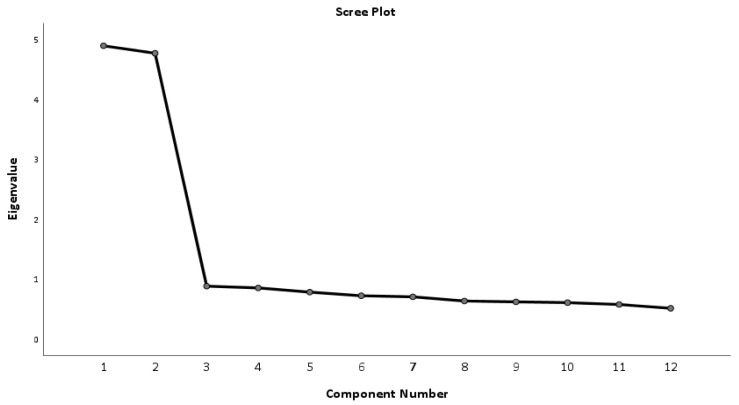
Scree plot (dehumanization scale).

CVR results showed that 100% (n = 12) of items were acceptable. The Bartlett Test of Sphericity was 1798.24 (*p* < 0.001). The Kaiser–Meyer–Olkin measure of sampling adequacy was 0.882, showing that the data are suitable for factor analysis. The 12 items were analyzed via the Principal Component extraction method, using a Varimax rotation. The analysis resulted in a model of two factors (Figure 1) that explains, based on the initial solution, 58.35% of the total variance. This percentage of the total dispersion is 58.31% if we take standardized values. We appear to have strong evidence for a two-factor model that included all 12 items (Table 4). Questions 1–6 load on factor 2 (animalistic dehumanization) and questions 7–12 on factor 1 (mechanistic dehumanization). Both mechanistic dehumanization and animalistic dehumanization had Cronbach’s alpha equal to 0.86.

#### 3.1.2. Mechanistic Self-Dehumanization Scale

**Figure 2:**
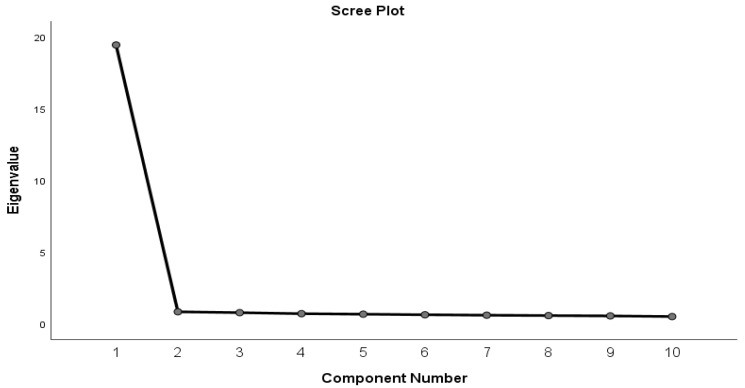
Scree plot (self-dehumanization scale).

CVR results showed that 100% (n = 10) of items were acceptable. The Bartlett Test of Sphericity was 4043.93 (*p* < 0.001). The Kaiser–Meyer–Olkin measure of sampling adequacy was 0.976, showing that the data were suitable for factor analysis. The 10 items were analyzed via the Principal Component extraction method, using a Varimax rotation. The analysis resulted in a model of one factor (Figure 2) that explains, based on the initial solution, 77.0% of the total variance. This percentage of the total dispersion was 76.7% if we take standardized values. We appear to have strong evidence for a single-scale factor model that included all 10 items (Table 5) with Cronbach’s alpha equal to 0.966.

#### 3.1.3. Means Score of the Dehumanization and Self-Dehumanization

**Figure 3:**
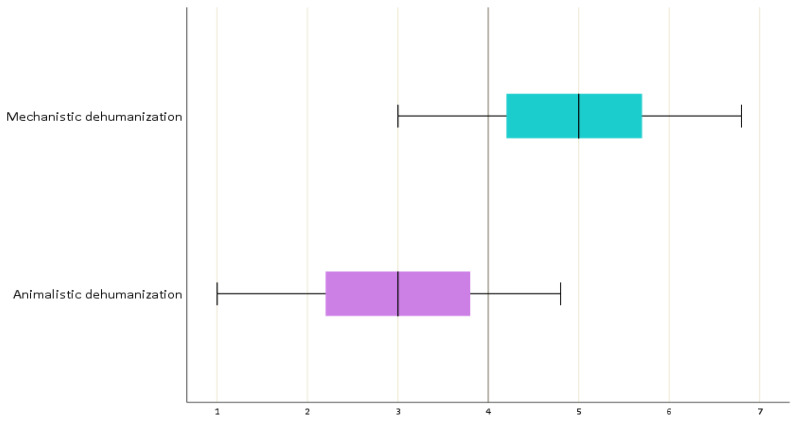
Box plot showing the hetero-dehumanization scale.

**Figure 4:**
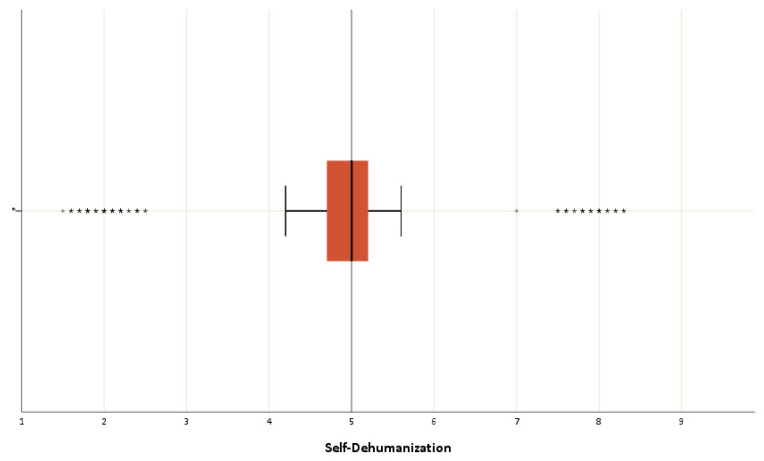
Box plot showing the mechanistic self-dehumanization scale. * Extreme values of the analysis.

The mean score of the mechanistic dehumanization subscale was calculated as 4.98 ± 0.90. The mean score of the animalistic dehumanization subscale was calculated as 3.02 ± 0.89 (Figure 3). The mean score of the mechanistic self-dehumanization scale was calculated as 4.83 ± 1.39 (Figure 4).

Table 6 depicts no association between socio-demographic and occupational factors and the mechanistic dehumanization and animalistic dehumanization subscales. A significant relationship was found between the self-dehumanization scale and years of work in the department (more years correspond to a higher scale score, *p* = 0.009). Attendance of a psychotherapy session (individuals who have attended psychotherapy sessions are associated with higher scores on the scale, *p* = 0.031). Finally, Table 7 depicts no association between the marital status of the participants and the mechanistic, animalistic or mechanistic self-dehumanization subscales. 

## 4. Discussion

In recent years, the scientific community has shown an increased interest in the phenomenon of dehumanization, which has extensively been examined in the field of social psychology [1,30,31,33,41,42,43,44]. It is well known that healthcare professionals, and especially doctors and nurses, frequently have to face this particularly difficult task. Since health science is a human-centered profession, this dynamic scientific field requires that professionals show a lot of passion and love to face challenges both psychologically and physically and maintain their integrity over time.

However, dehumanization does not always come from immoral behavior or social rejection. Everyday dehumanization is likely to be unintentional, arising from a variety of factors usually related to structural and organizational aspects of health care [10,30,45]. Unfortunately, multiple difficulties in public hospitals—workplaces, the nature of the profession, as well as the lack of staff and motivation—increase physical and psychological fatigue, which easily leads to real burnout [10]. We would add that elements of personality, empathy, technology, and the external pressures of everyday life are factors contributing to this behavior among professionals. In this context, dehumanization is a complex psychological defense that Ironically helps healthcare providers cope with and survive multiple unpleasant and debilitating personal and professional conditions [11]. Therefore, it is an emotional regulation strategy used by health care professionals [10], as it mentally gives them a shield to alleviate the anxiety and feelings of guilt that may arise from causing pain [9,46]. Furthermore, an empirical study of the effect of dehumanization in the era of computerized information was done through Amazon Mechanical Turkish. The results showed that there appears to be a negative effect of the computer on the doctor-patient relationship and the resulting patient care [47].

The result of all the above observations indicates that in medical environments, multiple behavioral events offend the dignity of patients. Some healthcare professionals use inappropriate communication styles, using baby talk, when speaking to elders, while others display various forms of aggressive behavior when interacting with the mentally ill [48,49,50].

The main objective of this study was to validate measures for the assessment of both types of dehumanization, namely animalistic and mechanistic dehumanization, in the field of healthcare, where dehumanization is a rampant phenomenon [2,12]. To the best of our knowledge, there was a lack of validated instruments for measuring dehumanization among healthcare professionals in Greece but also globally, a gap that this research aims to fill. In fact, the lack of validated instruments can lead to under-investigation of the phenomena and research initiative suspension [51]. Utilizing the validated questionnaires in our research both for dehumanization and self-dehumanization will enable researchers to systematically investigate and establish correlations between this phenomenon and other variables, thereby bridging gaps in the existing literature. Also, the thoughtful evaluation of the dehumanization phenomenon can deepen health professionals’ understanding of its complexities and guide focused interventions.

Measuring dehumanization among healthcare professionals is essential because it helps identify attitudes and behaviors that may undermine patient care and well-being. Dehumanization can lead to reduced empathy, poorer patient-provider communication, and ultimately lower quality of care [9,33,34]. By quantifying these tendencies, healthcare organizations can implement targeted interventions to promote compassion, improve patient outcomes, and foster a more supportive and ethical healthcare environment. Understanding and addressing dehumanization can also contribute to the mental health and job satisfaction of healthcare providers by emphasizing the humane and respectful treatment of patients.

Results of the present study revealed that both validated scales are psychometrically sound and theoretically valid measures of dehumanization and self-dehumanization. Factor analysis of the dehumanization scale resulted in a two-factor solution consisting of six items per factor and interpreting 58.35% of the total variance. The two factors were namely mechanistic and animalistic dehumanization [2]. This result is in agreement with the theoretical framework of dehumanization proposed by Haslam (2006) that distinguishes those two forms of dehumanization. Furthermore, factor analysis resulted in a one-factor solution for the self-dehumanization scale, interpreting 77.0% of the total variance in agreement with the Bastian (2014) theoretical model [52] and reinforcing previous studies that are considering and investigating self-dehumanization with unidimensional scales. All items in both scales exhibited excellent factor loadings all above the threshold value of 0.30, indicating an acceptable structural validity of the validated instruments [53]. Factor analysis was used to explore the questionnaire’s validity, revealing clear dimensions that accurately represent the underlying concepts we aimed to measure. The high factor loadings indicate that each question effectively contributes to its respective dimension, confirming the questionnaire’s validity.

In addition, according to our results, Cronbach’s alpha indicator values were acceptable. More specifically, the reported values were 0.855 for the “mechanistic dehumanization” subscale and 0.858 for the “animalistic dehumanization” subscale, whereas the value was equal to 0.966 for self-dehumanization. The mentioned values were above the threshold of 0.70, indicating good reliability for the values above 0.80 and excellent reliability for those above 0.90 [54]. This means that the questions consistently measure the intended concepts, making our questionnaire a dependable tool for research.

The statistical study’s results showed that doctors and nurses mechanistically dehumanized patients at a moderate to a high level and also dehumanized themselves at a moderate level. These results are consistent with the existing literature. According to the correlations conducted in our research, no socio-demographic and occupational factors were found to be associated with the mechanistic dehumanization and animalistic dehumanization subscales. A significant relationship was found between the self-dehumanization scale and (a) years of work in this department (more years correspond to a higher scale score; *p* = 0.009), and (b) attending a psychotherapy session (individuals who have attended psychotherapy sessions are associated with higher scores on the scale; *p* = 0.031). Furthermore, marital status was not found to be associated with any scale.

As aforementioned, more years of experience is a risk factor for increased levels of dehumanization for healthcare professionals. Prolonged exposure to high workloads, stressful environments, and emotionally challenging patient interactions can gradually diminish a healthcare professional’s sense of self-esteem, leading to feelings of burnout, emotional exhaustion, and a reduced ability to empathize with others. Additionally, although the finding that healthcare providers attending a psychotherapy session seems controversial, it may be reasonable considering that psychological distress and vulnerability, common reasons for seeking therapy, can contribute to the self-dehumanization or dehumanization of others. Also, the therapeutic process itself may lead to increased feelings of shame or inadequacy, feelings that can promote self-dehumanization or hetero-dehumanization as an outcome or a defensive reaction, respectively. The same effect can be triggered by the therapeutic environment, which can disclose identity conflicts and internal struggles, exacerbating experiences of dehumanization. A recent study carried out at the Sotiria Hospital in Athens, Greece, showed that healthcare professionals mechanically dehumanize hospitalized patients [30]. Another study found that nurses stigmatized and dehumanized patients with a psychiatric disorder more often compared to people without a mental illness. This occurrence might be related to the cultural stigma associated with mental illness, resulting in unfavorable prejudices and stereotypes. Additionally, the complexities and challenges of managing psychiatric disorders in healthcare settings, combined with potential gaps in mental health education and training, may contribute to elevated stress and frustration among healthcare providers, influencing their attitudes and behaviors towards patients [20].

Moreover, nurses tended to dehumanize patients more if they felt dehumanized by their supervisors and less if they had quality contacts [20]. This emphasizes the pivotal role of workplace dynamics and organizational culture that influence nurses’ attitudes and behaviors toward patient care. Establishing a supportive and respectful workplace atmosphere is paramount for both fostering an empathetic and compassionate environment for patients and bolstering the overall effectiveness and well-being of nurses.

Self-dehumanization is a phenomenon that can result either from being treated negatively by others or from one’s behavior when it harms others [1,2,33]. We would dare to say that the “offender” is also a “victim”. Their character and experiences led them to self-dehumanization and the dehumanization of others. Research studies have shown that self-dehumanization occurs most often when people feel isolated, powerless, or out of control. A literature review of several studies showed that self-dehumanization appears to be due to a sense of helplessness rather than a random change in mood or negative self-esteem [55]. In another study, it was shown that the suffering experienced by the person and unpleasant situations can cause the rejection of humanity both in ourselves and in others [1]. In a recent study in Greece, it was confirmed that mental health specialists and hospital workers consider themselves more human compared to the general population, attributing to themselves characteristics of human nature and uniqueness [33]. However, in some cases, self-dehumanization can encourage the individual to take social initiatives to restore humanity in the eyes of others and their self-concept [52], which can be a hope for reducing this phenomenon.

As mentioned earlier in the current study, the findings show that the longer someone works, the more they dehumanize themselves and, consequently, their patients. This highlights how healthcare professionals become exhausted, distance themselves from human suffering, and sometimes become insensitive in their efforts to protect themselves. Over time, individuals tend to view their work more detachedly, adopt a more cynical approach to situations and people, and ultimately, appear to self-dehumanize. Dehumanization may function as a defense mechanism that individuals develop over time/experience to cope with the challenges of the profession.

Another significant finding is that a notable relationship was found between the scale of self-dehumanization and attending psychotherapy sessions (individuals who have attended psychotherapy sessions are associated with higher scores on the scale). This is a paradoxical result. Perhaps one explanation is that those who undergo psychotherapy may consciously choose self-dehumanization as a defense mechanism that helps them cope. For example, they learn that taking emotional distance from the experiences they encounter offers them benefits that, in the long run, allow them to remain in their job. However, it is worth investigating whether dehumanization ultimately has positive outcomes in addition to negative ones.

To prevent staff burnout and the dehumanization of patients in healthcare settings, institutions must prioritize efforts to restore the well-being of their personnel.

Health sector leadership must study these phenomena, restore the trust of healthcare workers, and respond to their needs. Authorities must examine risk factors and protection measures for healthcare workers and develop corresponding treatment and prevention policies immediately. This includes recording updated healthcare needs at a national level, implementing permanent hires and appointments, rationally distributing healthcare personnel, and planning rotating schedules. Additionally, methodical training in mindfulness, expanding psychosocial support, and self-care for healthcare professionals (breaks in specially designed hospital areas, time with family and friends, and organized days off) is recommended. Furthermore, multi-level scientific psychiatric and psychological intervention programs within hospitals are suggested, such as self-disclosure, psychological debriefing, and discussion after distressing events (e.g., patient death), as well as group psychotherapy to resolve issues both on an individual level and for improved teamwork.

### Limitations

The present research faced some limitations. First of all, women outnumbered men in the sample, which is due to the nature of the profession as the majority of nurses are women. Secondly, the collected sample of 400 individuals, although large enough for statistical analysis, was not randomly selected. Finally, the cross-sectional study design limits the ability to establish causal relationships or assess changes over time.

The main strength of the present study is the establishment of two valid questionnaires intended to evaluate different aspects of dehumanization among healthcare professionals. Providing precise measures to evaluate dehumanizing actions and attitudes is one of the ways our research directly contributes to improving patient care and developing a more compassionate and empathic atmosphere within the healthcare system. These scales not only enhance research capacities in the area of studying dehumanization, but they also have practical use in directing interventions and training programs that are targeted at encouraging patient-centered care and professional ethics among healthcare workers.

## 5. Conclusions

Our study concluded with two valid scales that can be used to investigate the phenomenon of the dehumanization of patients and the self-dehumanization of health professionals. The statistical analysis revealed that health professionals were found to mechanistically dehumanize patients at a moderate to high level, while they also dehumanize themselves at a moderate level. Dehumanization appears to be highly relevant to several important psychosocial aspects of medical care, including quality of care, health inequity, health behaviors, and person-centered care, which poses the necessity for further investigation of this phenomenon. Future research through new questionnaires can explore the effectiveness of these interventions. Also, the thoughtful evaluation of this phenomenon can deepen our understanding of its complexities and provide guidance for focused interventions. The results can contribute to the creation of information and awareness programs and educational programs that will help doctors and nurses, as well as the students of these universities, to deal more humanely with their patients. Health professionals should be informed about the current situation, and healthcare policy-makers and stakeholders should take the necessary actions and measures to identify potential improvements, ensure accountability, and promote patient-centered care.

## Figures and Tables

**Table 1:** Characteristics of sample.

			Mean (SD)
Age		43.8 ± 10.5	
Working experience		9.2 ± 8.8	
		N	%
Biological sex	Male	103	25.7%
	Female	297	74.3%
Place of residence	Village	55	13.8%
	City <150,000 inhabitants	127	31.8%
	City >150,000 inhabitants	218	54.5%
Marital status	Unmarried	131	32.8%
	Married	237	59.3%
	Divorced	29	7.3%
	Widower	3	0.8%
Profession	Nursing	294	73.5%
	Medicine	106	26.5%
Annual income	≤20.000 €	343	85.8%
	>20.000 €	57	14.3%
	BSc	319	79.8%
Post-graduate degrees	MSc	66	16.5%
	PhD	15	3.7%
Labor Department	Ward	195	48.7%
	Unit	205	51.3%
Working hours	Circular timetable	298	74.5%
	Morning/afternoon schedule	102	25.5%
Have you attended any psychotherapy sessions?	Yes	66	16.5%
	No	334	83.5%

Mean (standard deviation); BSc: Bachelor of Science, MSc: Master of Science; PhD: Doctor of Philosophy degree.

**Table 2:** Dehumanization scale answers.

	Totally Disagree		2		3		4		5		6		Completely Agree	
	N	%	N	%	N	%	N	%	N	%	N	%	N	%
They are superficial people and have no depth.	41	10.3	92	23.0	131	32.8	88	22.0	45	11.3	2	0.5	1	0.3
They are open-minded people who can think clearly.	49	12.3	93	23.3	130	32.5	92	23.0	34	8.5	1	0.3	1	0.3
They are cold people and act like robots/machines.	46	11.5	90	22.5	130	32.5	91	22.8	40	10.0	1	0.3	2	0.5
They are people who feel warmth in their relationships with others.	36	9.0	95	23.8	123	30.8	102	25.5	40	10.0	3	0.8	1	0.3
They act as if they themselves are objects and not people.	38	9.5	92	23.0	133	33.3	89	22.3	45	11.3	1	0.3	2	0.5
They are emotional people, showing responsiveness and warmth.	45	11.3	85	21.3	133	33.3	94	23.5	40	10.0	3	0.8	0	0.0
They are rational and sensible people. They are smart.	2	0.5	1	0.3	42	10.5	89	22.3	130	32.5	88	22.0	48	12.0
They are people inferior to the human species, like animals.	1	0.3	2	0.5	40	10.0	81	20.3	152	38.0	76	19.0	48	12.0
They are refined and civilized people.	1	0.3	1	0.3	44	11.0	80	20.0	150	37.5	89	22.3	35	8.8
They have self-control.	1	0.3	2	0.5	48	12.0	62	15.5	151	37.8	87	21.8	49	12.3
They are uncivilized people, without good manners at all.	1	0.3	2	0.5	46	11.5	88	22.0	144	36.0	81	20.3	38	9.5
They act like adults and not like children (i.e., they are mature).	2	0.5	1	0.3	47	11.8	86	21.5	128	32.0	96	24.0	40	10.0

**Table 3:** Self-dehumanization scale answers.

	Totally Disagree		2		3		4		5		6		7		8		Totally Disagree	
	N	%	N	%	N	%	N	%	N	%	N	%	N	%	N	%	N	%
I make most of my decisions based on logic.	24	6.0	18	4.5	11	2.8	111	27.8	98	24.5	104	26.0	12	3.0	10	2.5	12	3.0
I don’t usually operate in an emotional way.	18	4.5	19	4.8	16	4.0	107	26.8	98	24.5	108	27.0	13	3.3	7	1.8	14	3.5
I feel that my actions are not based personal motives, but are due to automatisms.	7	1.8	12	3.0	34	8.5	118	29.5	134	33.5	61	15.3	34	8.5	0	0.0	0	0.0
I am not open to stimuli from the outside world, which are alien or unknown to me.	17	4.3	19	4.8	17	4.3	93	23.3	128	32.0	92	23.0	14	3.5	7	1.8	13	3.3
In my relationships with others, I like to express my feelings.	21	5.3	18	4.5	14	3.5	94	23.5	108	27.0	111	27.8	16	4.0	8	2.0	10	2.5
I often feel that I lack depth, that I am a bit superficial.	17	4.3	17	4.3	19	4.8	122	30.5	91	22.8	100	25.0	12	3.0	8	2.0	14	3.5
I feel warmth in my relationships with others.	16	4.0	19	4.8	18	4.5	104	26.0	111	27.8	98	24.5	12	3.0	11	2.8	11	2.8
I am open to every new experience.	21	5.3	22	5.5	10	2.5	107	26.8	103	25.8	103	25.8	10	2.5	14	3.5	10	2.5
Sometimes I work like a machine: without thinking about it, I do certain things like an automaton.	18	4.5	17	4.3	18	4.5	98	24.5	104	26.0	111	27.8	11	2.8	12	3.0	11	2.8
I believe that most of my actions and choices in life come from my own autonomous intentions and preferences.	18	4.5	13	3.3	22	5.5	104	26.0	106	26.5	103	25.8	13	3.3	14	3.5	7	1.8

**Table 4:** Rotated component matrix (dehumanization scale).

	Raw		Rescaled	
	Component		Component	
	1	2	1	2
DS-1		0.894		0.757
DS-2		0.872		0.749
DS-3		0.923		0.775
DS-4		0.902		0.774
DS-5		0.876		0.744
DS-6		0.896		0.766
DS-7	0.942		0.782	
DS-8	0.837		0.717	
DS-9	0.842		0.745	
DS-10	0.944		0.789	
DS-11	0.906		0.781	
DS-12	0.918		0.768	

Extraction Method: Principal Component Analysis. Rotation Method: Varimax with Kaiser Normalization.

**Table 5:** Rotated component matrix (self-dehumanization scale).

	Raw	Rescaled
	Component	Component
	1	1
SDS-1	1.478	0.882
SDS-2	1.429	0.873
SDS-3	1.045	0.828
SDS-4	1.417	0.888
SDS-5	1.415	0.873
SDS-6	1.417	0.872
SDS-7	1.410	0.884
SDS-8	1.486	0.896
SDS-9	1.432	0.884
SDS-10	1.366	0.871

Extraction Method: Principal Component Analysis. SDS = Self-Dehumanization scale.

**Table 6:** Correlations.

		Age	Biological Sex	Place of Residence *	Marital Status	Profession	Annual Income	Postgraduate Degrees	Labor Department	Years of Work in This Department	Working Hours	Have You Attended any Psychotherapy Sessions?
Mechanistic dehumanization	Correlation	−0.060	−0.013	−0.001	0.019	−0.055	0.017	0.102	−0.072	0.031	−0.001	−0.007
	Sig. (two-tailed)	0.230	0.802	0.981	0.699	0.275	0.729	0.363	0.149	0.538	0.986	0.888
	N	400	400	400	400	400	400	81	400	400	400	400
Animalistic dehumanization	Correlation	−0.075	−0.016	−0.019	0.015	−0.050	0.024	0.097	−0.067	0.008	0.013	−0.017
	Sig. (two-tailed)	0.132	0.755	0.699	0.765	0.316	0.637	0.390	0.184	0.880	0.795	0.739
	N	400	400	400	400	400	400	81	400	400	400	400
Self-dehumanization	Correlation	0.035	0.087	0.061	−0.011	−0.078	−0.013	−0.127	0.023	0.131	−0.060	0.108
	Sig. (two-tailed)	0.483	0.081	0.225	0.822	0.120	0.789	0.259	0.653	**0.009**	0.230	**0.031**
	N	400	400	400	400	400	400	81	400	400	400	400

* Spearman Correlation. Statistically significant correlations (*p* < 0.05) are highlighted in bold font.

**Table 7:** Analysis of variance (ANOVA) between marital status and scales.

		Sum of Squares	df	Mean Square	F	Sig.
Mechanistic dehumanization	Between Groups	0.944	3	0.315	0.387	0.762
	Within Groups	321.487	396	0.812		
	Total	322.431	399			
Animalistic dehumanization	Between Groups	6.041	3	2.014	2.556	0.055
	Within Groups	312.016	396	0.788		
	Total	318.057	399			
Mechanistic self-dehumanization	Between Groups	5.985	3	1.995	1.034	0.378
	Within Groups	764.335	396	1.930		
	Total	770.320	399			

## Data Availability

The data that support the findings of this study are available from the corresponding author upon reasonable request.

## Public Involvement Statement

No public involvement in any aspect of this research.

## Guidelines and Standards Statement

This manuscript was drafted against The Strengthening the Reporting of Observational Studies in Epidemiology (STROBE) Statement: guidelines for reporting observational studies [56].

## Appendix A

### Appendix A.1. Dehumanization Scale

“How would you generally describe the patients hospitalized at the hospital where you work? Circle only one number from 1 (=strongly disagree) to 7 (=strongly agree) depending on the extent to which you agree or disagree with the content of each statement.

1) **They are superficial people and have no depth.** **I strongly disagree 1 2 3 4 5 6 7 I strongly agree**
2) **They are open-minded people who can think clearly** **I strongly disagree 1 2 3 4 5 6 7 I strongly agree**
3) **They are cold people and act like they are robots/machines.** **I strongly disagree 1 2 3 4 5 6 7 I strongly agree**
4) **They are people who feel warmth in their relationships with others.** **I strongly disagree 1 2 3 4 5 6 7 I strongly agree**
5) **They act as if they themselves are objects and not people.** **I strongly disagree 1 2 3 4 5 6 7 I strongly agree**
6) **They are emotional people. Showing responsiveness and warmth.** **I strongly disagree 1 2 3 4 5 6 7 I strongly agree**
7) **They are rational and sensible people. They are smart.** **I strongly disagree 1 2 3 4 5 6 7 I strongly agree**
8) **They are persons inferior to the human species. Like animals.** **I strongly disagree 1 2 3 4 5 6 7 I strongly agree**
9) **They are refined and civilized people.** **I strongly disagree 1 2 3 4 5 6 7 I strongly agree**
10) **They have self-control.** **I strongly disagree 1 2 3 4 5 6 7 I strongly agree**
11) **They are uncivilized people. Without good manners at all.** **I strongly disagree 1 2 3 4 5 6 7 I strongly agree**
12) **They act like adults and not like children (i.e., they are mature).** **I strongly disagree 1 2 3 4 5 6 7 I strongly agree**

The answers to sentences 2, 4, 6, 7, 9, 10, and 12 are reversed so that all 12 sentences have the same conceptual direction.

## Appendix B

### Appendix B.1. (Mechanistic) Self-Dehumanization Scale

There are no right or wrong answers. We are interested in your personal opinion. In the following questions, circle the number that best corresponds to your own viewpoint.

1) **I make most of my decisions based on logic.** **I strongly disagree 1 2 3 4 5 6 7 8 9 I strongly agree**
2) **I don’t usually operate in an emotional way.** **I strongly disagree 1 2 3 4 5 6 7 8 9 I strongly agree**
3) **I feel that my actions do not have personal motivations. but obey automatisms.** **I strongly disagree 1 2 3 4 5 6 7 8 9 I strongly agree**
4) **I am not open to stimuli from the outside world. which are foreign or unknown to me.** **I strongly disagree 1 2 3 4 5 6 7 8 9 I strongly agree**
5) **In my relationships with others, I like to express my feelings.** **I strongly disagree 1 2 3 4 5 6 7 8 9 I strongly agree**
6) **I often feel that I lack depth. that I am a bit superficial.** **I strongly disagree 1 2 3 4 5 6 7 8 9 I strongly agree**
7) **I feel warmth in my relationships with others.** **I strongly disagree 1 2 3 4 5 6 7 8 9 I strongly agree**
8) **I am open to every new experience.** **I strongly disagree 1 2 3 4 5 6 7 8 9 I strongly agree**
9) **Sometimes I work like a machine: without thinking about it. I do certain things** **things like automatic.** **I strongly disagree 1 2 3 4 5 6 7 8 9 I strongly agree**
10) **I believe that most of my actions and choices in life come from my own autonomous intentions and preferences.** **I strongly disagree 1 2 3 4 5 6 7 8 9 I strongly agree**

The answers to sentences 5, 7, 8, and 10 are reversed so that all 10 sentences have the same conceptual direction.
